# Self-Harm Behaviors, Suicide Attempts, and Suicidal Ideation in a Clinical Sample of Children and Adolescents with Psychiatric Disorders

**DOI:** 10.3390/children10040725

**Published:** 2023-04-14

**Authors:** Elena Predescu, Roxana Sipos

**Affiliations:** Department of Neuroscience, Psychiatry and Pediatric Psychiatry, “IuliuHatieganu” University of Medicine and Pharmacy, 400460 Cluj-Napoca, Romania; predescu.elena@umfcluj.ro

**Keywords:** suicidal attempt/ideation, self-harm behavior, psychiatric disorders, children and adolescents

## Abstract

Suicidal ideation and self-harm behaviors have been found to be important risk factors for suicide. The aim of this study was to explore the rates of psychiatric disorders among different groups of patients with suicidal ideation, suicide attempts, and non-suicidal self-harm behaviors and to identify the associated socio-demographic and clinical variables. We conducted a cross-sectional study with emergency-admitted patients presenting with non-suicidal self-harm behaviors, suicide attempts, or suicidal ideation to the emergency room of the Child and Adolescent Psychiatry Clinic in Cluj-Napoca, Romania. Data were collected from the patients’ charts using a questionnaire that contained socio-demographic and clinical variables. A total of 95 patients aged between 6 and 18 years were included in the study. Ingesting medication and cutting were the most frequently used methods to attempt suicide. Depression and mixed affective and conduct disorders were the diagnoses most commonly associated with suicidal behavior. Girls with depressive symptoms were more probable to have suicide attempts than boys, and girls with depressive symptoms and behavioral problems registered more self-harm behaviors. Further research should systematically examine the relationship between self-harm behaviors and suicide attempts and the profile of patients at risk of future suicide attempts.

## 1. Introduction

Self-harm behaviors, suicide attempts, and suicidal ideation are major global mental health problems due to their increased frequency and severity. Suicidal behavior and self-harm behaviors are two distinct concepts; although they may share some similarities, the relationship between them is complex. Suicidal behavior refers to any act with the intent to end one’s own life, such as attempting suicide, making a suicide plan, or expressing suicidal thoughts or feelings. On the other hand, self-harm behaviors, also known as non-suicidal self-injuries (NSSIs), refer to any deliberate act of self-injury or self-harm without the intent to die. This can include cutting, burning, hitting oneself, or any other behavior that results in physical harm. NSSIs are often used as a coping mechanism to deal with emotional pain, stress, or trauma and are more frequent than suicide attempts [1,2,3], with the prevalence of self-harm behaviors being almost double that of suicide attempts. Because these behaviors have different outcomes and may also have different backgrounds, non-suicidal self-injury and suicidal behavior are classified as distinct clinical phenomena in the *DSM-5* [4] (see “Conditions for Further Study”). Non-suicidal and suicidal self-harm often co-occur, especially in clinical samples [5,6], and NSSI behaviors may prove to be a strong predictor for future suicidal attempts [7,8,9,10]. Considering that approximately 12.1% of adolescents report suicidal ideation and 4.1% of adolescents have at least one suicide attempt by the age of 18, the presence of NSSI and its implication should be more carefully assessed, especially in clinical settings [11,12].

Overall, suicide rate in childhood (<10 years) has been found to be low but increases dramatically with puberty [13]. The frequency of suicidal behaviors in children and adolescents varies depending on gender, psychiatric disorders, socio-economic and cultural factors, and environmental and genetic factors [10,14]. Some studies estimate that suicidal ideation and suicide attempts are more common in females, but in men, suicide attempts result in more frequent deaths [15].

There is a strong association between suicidal ideation, suicide attempts, self-harm behaviors, and mental health problems in adolescents (MHPs). Adolescents experiencing MHPs are at a higher risk of engaging in these behaviors [16,17,18,19]. Most children and adolescents presenting with NSSI, suicidal ideation, and/or suicide attempt meet the diagnostic criteria for at least one psychiatric diagnosis. Research has shown that depression, anxiety, and post-traumatic stress disorder (PTSD) are common mental health problems associated with suicidal ideation, suicide attempts, and self-harm behaviors in adolescents. Other mental health problems that have been linked to these behaviors include attention-deficit/hyperactivity disorder (ADHD), oppositional defiant disorder (ODD), and conduct disorder (CD) [20].

Very few studies examined the possible connection between conduct disorder (CD) symptoms and self-harm behaviors in children and adolescents [21,22,23,24]. Some of these studies found significant positive associations between CD or oppositional defiant disorder (ODD) symptoms and deliberate self-harm behaviors [21]. High prevalence rates of NSSI were registered among patients with attention deficit/hyperactivity disorder (ADHD) and conduct disorder, depending on the participants’ gender and alcohol dependence [25,26], and reached a prevalence rate of 92.0% among male offenders with antisocial personality disorder [27]. Peers’ self-harm behaviors can be “contagious” in children and adolescents, with research on this topic suggesting that, for some individuals, exposure to self-harm behaviors through peers and/or the media may contribute to the onset and maintenance of these behaviors [28].

While suicide attempts and self-harm behaviors may have shared clinical characteristics [22,29] in patients diagnosed with borderline personality disorder [23], depression, anxiety [30], and alcohol dependence [27], most publications have focused on the distinguishing factors between them. Children and adolescents who engage in self-injurious behaviors with and without suicidal intent may differ in the frequency and methods of self-injury, comorbid symptoms, and presence of personality disorders [23]. Some studies have suggested that externalizing symptoms are associated with self-harm behaviors [31,32], while internalizing symptoms may play a greater role in suicidality [29,31,32]. Conversely, other studies have found higher levels of both internalizing and externalizing symptoms in individuals who have engaged in both suicide attempts and non-suicidal self-injuries [30,33].

The purpose of this study was to explore the rates of psychiatric disorders among different groups of patients with suicidal ideation, suicide attempts, and non-suicidal self-harm behaviors and to identify the associated socio-demographic and clinical variables. Based on previous studies stating that internalizing symptoms are more present in people attempting suicide, we hypothesized that a higher prevalence of affective and anxiety disorders would be found in patients with suicidal ideation and suicide attempts and that patients with non-suicidal self-harm behaviors would have a higher rate of externalizing disorders.

## 2. Materials and Methods

### 2.1. Participant Selection

We selected a clinical sample of 194 patients, aged between 6 and 18 years, with emergency admissions in the Child and Adolescent Psychiatry Clinic in Cluj-Napoca. After applying the inclusion and exclusion criteria, 95 patients were included in the study. The inclusion criteria were boys or girls aged 6–18; presenting with self-harm behaviors, suicide attempts, or suicidal ideation; with emergency admission for assessment or treatment; and with agreement from the child/adolescent and caregiver that their medical data could be used for research purposes. We excluded patients with other problems, those who received services in out-patient settings, and those who did not agree to participate. All patients were diagnosed using the 10th revision of the *International Statistical Classification of Diseases and Related Health Problems* (ICD 10) based on clinical interviews conducted by a trained child psychiatrist. Of the included patients, 45 (47.4%) were male and 50 (52.6%) were female, with a mean age of 14.58 (SD = 2.07). The patients were from various counties, had diverse socioeconomic backgrounds, and were all Caucasian.

This study was conducted in accordance with the laws concerning the conduct of clinical trials, as stated in the Helsinki Declaration of Human Rights. The patients and their parents signed the informed consent, agreeing that their medical data could be used for research purposes. The data were used according to the regulation regarding the privacy and protection of participants’ identity. We obtained the approval of the Emergency Clinical Hospital for Children Cluj-Napoca Clinical Trials Quality Assurance Commission, 45SC/11.03.2016.

### 2.2. Variables

This study was retrospective, and the data were collected from the patients’ medical records by two independent raters, with discrepancies being discussed and resolved by agreement between the raters. The data included socio-demographics, family environment, academic achievements, socio-economic level, family history and personal background, psychiatric diagnosis, treatment, suicide attempt methods, number of attempts, and reason for admission. The retrospective nature of the study allowed us to assess separately, for every patient, suicide attempt (as suicidal self-injury), self-harm behaviors (as self-inflicted injuries, but without the intent to die), and suicidal ideation (as being present or not present).

The variables describing the patients’ characteristics included age, gender, environment (defined as rural or urban), personal history of psychiatric disorders (defined as affective disorders, conduct disorders, ADHD, emotional disorders, substance use disorder, and other disorders), personal history of chronic somatic disorders (defined as being present or not), school performance (defined as poor, average, good, or very good), negative life events (categorized as death of a parent, death of a close person, divorced or separated parents, parents working abroad, school failure, and conflicts with parents or peers), and previous psychotherapy or pharmacological treatment (defined as being present or not).

The variables describing family characteristics included parents’ ages, marital status (categorized as married, single, or divorced/separated/widowed), employment status (categorized as unemployed or employed), educational level (categorized as graduating 8th grade, high school, or university), family conflicts (defined as being present or not) and family history of psychiatric disorders (categorized as substance use disorder, depression, schizophrenia, bipolar disorder, anxiety, or others). Socioeconomic status was categorized as low, medium, good, or very good.

### 2.3. Statistical Analysis

For data analysis, we utilized version 17 of the Statistical Package for the Social Sciences (SPSS). The patients’ characteristics were presented as means and standard deviations (SD) for continuous variables and as numbers (%) for categorical variables, for each category assessed (suicidal ideation, suicide attempt, and self-harm behaviors). We conducted *t*-tests for independent samples for each continuous variable and chi-squared tests for each categorical variable to assess the association between each of these variables and the categories of suicide attempt, suicidal ideation, and self-harm behaviors. We also used t-tests to examine gender and living environment differences. Additionally, we performed logistic regressions to predict suicidal ideation, self-harm behaviors, and suicide attempt from the interaction effects of sex, environment, and diagnosis. All tests were conducted at a significance level of 5%, with a 95% confidence interval (CI).

## 3. Results

### 3.1. Participant Socio-Demographic Characteristics

This study included 95 patients emergency admitted for self-harm behaviors, suicide attempts, or suicidal ideation, out of which 45 (47.4%) were male and 50 (52.6%) were female, resulting in a boy-to-girl ratio of 0.9:1. The mean age of boys and girls did not show a significant difference, with a mean age of 14.48 (SD = 2.52) for boys and a mean age of 14.68 (SD = 1.57) for girls (t = −0.44, *p* = 0.65).

A total of 89 (93.68%) participants attended secondary or high school, and 40 (42.1%) reported poor school results. A total of six (6.31%) participants did not attend school. Most of the participants (88; 92.6%) were living with their parents, siblings, or other relatives. A total of 30 (31.6%) participants had poor socioeconomic status, and almost half of them (49.5%) reported negative life events (death of a parent—7.4%, death of a close person—3.2%, divorced or separated parents—5.3%, parents working abroad—5.3%, school failure—6.3%, and conflicts with parents or peers—8.4%) and family conflicts. A total of 33 (34.7%) participants had a family history of psychiatric disorders: drug or alcohol abuse at 17.9%, depression at 8.4%, and anxiety at 1.1% (see Table 1).

### 3.2. Methods of Suicide Attempt and Clinical Diagnosis

A total of 17 (53.1%) participants attempted suicide by medication overdose, 11 (34.3%) by cutting their wrists, and the remaining 4 (12.5%) used one of the following two methods: hanging and ingesting toxic substances. Almost all the patients that attempted suicide (*n* = 32) also had previous NSSI and reported active suicidal ideation. In total, 46 (48.4%) patients had one previous suicide attempt, and 8.4% had at least 2 attempts. Of the patients included in this study, more than half (56.8%) received more than one clinical diagnosis, with 14.7% having two comorbidities and 6.3% having three. The most prevalent diagnosis among the sample was major depressive disorder (MDD) (86.3%), followed by behavioral problems (CD, ODD, or ADHD) which were diagnosed in 51.6% of the patients. Other disorders, such as anxiety disorders, tics, or eating disorders, were diagnosed in 13.7% of the patients. About 20% of the patients did not receive pharmacological therapy, while 78.9% of them were prescribed different treatments, including neuroleptics in 23.2% of the cases, selective serotonin reuptake inhibitors (SSRI) in 31.6% of the cases, mood stabilizers in 10.5%, benzodiazepines (BZD) in 5.3%, and atomoxetine in 8.4% of the cases. Around 32.6% of the patients received monotherapy, while 44.2% received a combination of two pharmacological treatments, such as mood stabilizers in combination with neuroleptic, SSRI and BZD, or SSRI and neuroleptic.

The most commonly occurring diagnosis for suicidal ideation was major depressive disorder, which was present in 42.2% of the boys and 38% of the girls included in the study. Self-harm behaviors were more present in patients diagnosed with mixed affective and conduct disorders (17.8% of the boys and 34% of the girls) and major depressive disorder (17.8% of the boys and 26% of the girls). As presented in Table 2, self-harm behaviors were significantly more frequent in girls than in boys, and when behavioral problems were added to depressive symptoms, the percentages increased, although only in girls. Similarly, suicide attempts were more frequent in girls (18% to 20%) than in boys (6.7% to 13.3%) for both diagnoses; however, for boys, the rate of suicide attempts was higher in those with depressive symptoms and without behavioral problems. There were no significant differences observed between the genders in relation to suicidal ideation, but girls with depressive symptoms had a higher probability of having suicide attempts compared to boys (18% vs. 13.3%, chi-square = 5.00; *p* = 0.02), and girls with both depressive symptoms and behavioral problems were more likely to exhibit self-harm behaviors than boys (34% vs. 17.8%, chi-square = 3.97; *p* = 0.04). No significant differences were found based on living environment in terms of principal diagnoses and clinical variables. However, when considering the entire sample, the patients from rural areas had a higher probability of experiencing suicidal ideation than those from urban areas (chi-square = 6.40; *p* = 0.01) (see Table 2).

We also ran several logistic regressions in order to detect interaction effects of environment, psychiatric diagnosis, and sex that could explain significantly more variance in the clinical variables than the predictors alone. Neither suicidal ideation, self-harm behaviors, nor suicide attempts could be predicted by the third- or second-order interactions, that is, sex-by-diagnosis-by-environment interaction (suicidal ideation, chi-square = 5.93, *p* = 0.051; self-harm behaviors, chi-square = 1.68, *p* = 0.43; and suicide attempts, chi-square = 0.27, *p* = 0.87), sex-by-diagnosis interaction (suicidal ideation, chi-square = 1.05, *p* = 0.59; self-harm behaviors, chi-square = 2.75, *p* = 0.25; and suicide attempts, chi-square, *p* = 4.21, *p* = 0.12), environment-by-diagnosis interaction (suicidal ideation, chi-square = 1.87, *p* = 0.39; self-harm behaviors, chi-square = 1.41, *p* = 0.49; and suicide attempts, chi-square, *p* = 1.02, *p* = 0.6), and sex-by-environment interaction (suicidal ideation, chi-square = 0.82, *p* = 0.36; self-harm behaviors, chi-square = 0.44, *p* = 0.5; and suicide attempts, chi-square, *p* = 2.23, *p* = 0.13).

The odds of having a suicide attempt for the studied sample was 0.36 (95% CI of 0.14, 0.89), with a risk of 0.56 in boys (95% CI of 0.32, 0.98) and 1.57 in girls (95% CI of 1.07, 2.22). Similarly, the odds of presenting self-harm behaviors for the studied sample was 0.41 (95% CI of 0.18, 0.94), with a risk of 0.63 in boys (95% CI of 0.41, 0.97) and 1.53 in girls (95% CI of 1.00, 2.34). Additionally, the odds of having suicidal ideation in the studied sample was 0.25 (95% CI of 0.08, 0.76), with a risk of 0.60 for the patients living in urban areas (95% CI of 0.43, 0.84) and 2.35 for those living in rural areas (95% CI of 1.05, 5.23).

## 4. Discussion

The main purpose of this study was to examine the rates of different psychiatric disorders in specific groups of high-risk patients, who were admitted with suicidal ideation, suicide attempts, and non-suicidal self-harm behaviors, and to identify the associated socio-demographic and clinical variables. There is a well-established association between suicidality and self-harm behaviors. Many adolescents who engage in self-harm behaviors, such as cutting or burning themselves, also report suicidal thoughts or behaviors, with the prevalence rates increasing in recent years [34,35]. The NSSI prevalence is reported to be higher than the prevalence of suicidal behaviors [1,2,3], a situation that was also found in our study, with the prevalence of self-harm behaviors being almost double than that of suicide attempts. Adolescents who engage in NSSI are at a greater risk of attempting and completing suicide, particularly if they have a history of multiple suicide attempts or engage in more severe forms of self-harm. Research suggests that females may be more susceptible to suicidal ideation and attempts [15], but our study did not find any significant gender differences in suicidal ideation. However, we did find that girls with depressive symptoms were more prone to attempting suicide than boys, and girls with both depressive symptoms and behavioral problems were more likely to engage in self-harm behaviors than boys.

A particularity in our sample was the fact that almost all admissions (except one) for suicide attempts were associated with pre-existing NSSI. In a study with both clinical and community samples, Duarte et al. found that while 99.1% adolescents with NSSI reported suicide attempts, only one adolescent without NSSI reported a suicide attempt [36]. Several studies indicate that adolescents who engage in both NSSI and suicide attempts have increased rates of psychiatric disorders, notably major depression and post-traumatic stress disorder (PTSD), as well as more severe symptoms, such as suicidal ideation, depression, hopelessness, and higher impulsivity. Furthermore, family dysfunction and child maltreatment appear to be more prevalent among adolescents who report both NSSI and suicide attempts [7,11]. Our results were similar: more than half of the patients included in the study (56.8%) had more than one diagnosis, with 14.7% having two comorbidities and 6.3% having three. Most of the patients (86.3%) were diagnosed with major depressive disorder (MDD), and half of them had behavioral problems (CD, ODD, or ADHD). In a study with a non-suicidal self-injury group, Nock et al. [6] discovered that 62.9% of the participants had an externalizing disorder, while 51.7% had an internalizing disorder, and 70% of the participants had at least one suicide attempt, with 55% having multiple attempts. In our sample, 60% of the NSSI patients were admitted due to an actual suicide attempt, while nearly half of the participants (48.4%) had a history of previous suicide attempts, and 8.4% had attempted suicide at least twice.

Externalizing symptoms, such as aggression, impulsivity, and substance abuse, have been found to contribute to suicidality in adolescents. Research has consistently shown that adolescents who exhibit these symptoms are at an increased risk for suicide attempts and completed suicide. Aggression, for example, has been found to be a significant predictor of suicidal behaviors in adolescents. Adolescents who engage in physical fights or verbal arguments, threaten or intimidate others, or destroy property are more likely to have suicidal ideation, attempt suicide, or die by suicide [37]. Similarly, impulsivity is another externalizing symptom that has been linked to suicidality in adolescents. Impulsive behaviors, such as engaging in risky activities, substance abuse, or reckless driving, have been found to increase the risk of suicidal ideation and attempts [38]. Hurtig et al. found that adolescents diagnosed with ADHD were more likely to report suicidal ideation and self-harm compared to those without the disorder [30]. Another study identified female gender and depression/anxiety as factors that were linked to both suicidal ideation and self-harm. On the other hand, behavioral disorder, substance use, and family factors, such as living in a disorganized or financially challenged family, were only associated with NSSIs, and not with suicidal ideation or attempts [20]. These findings suggest that multiple factors contribute to suicidal ideation and self-harm in adolescents, and that the risk varies depending on the specific factors involved. 

The high rates of internalizing and externalizing problems in our sample and the presence of both suicidal ideation and previous NSSI in almost all patients included in the study with actual suicide attempt could indicate that the association between NSSI and suicidal ideation is highly predictive of future suicidal attempts. Duarte et al. have reported similar findings, stating that suicidal ideation and diversity of NSSI are significant predictors of suicide attempts when included in a prediction model [36]. This suggests that individuals who engage in both suicidal ideation and NSSI are at a higher risk of attempting suicide, highlighting the critical need for appropriate identification, intervention, and treatment for individuals who exhibit suicidal ideation and NSSI behaviors. A longitudinal study conducted in Canada found that depressive symptoms were frequently linked to both passive and serious suicidal ideation, while externalizing issues, such as conduct problems, oppositional and/or defiant behaviors, and attentional and/or hyperactivity symptoms, were associated with suicide attempts. This study also revealed that conduct problems were the most reliable predictor of suicide attempts across various developmental stages, even after considering the impact of depressive symptoms, with stronger associations observed among younger adolescents [20]. Previous longitudinal studies have also shown a relationship between both internalizing and externalizing problems and the risk for suicidal behaviors [39,40], highlighting the need for comprehensive assessment and intervention strategies that address both internalizing and externalizing problems in adolescents considered at risk of suicide attempt. Two comorbid profiles, depression associated with anxiety and depression associated with disruptive disorder, have been found to be accounted for the most important risk [18].

Liu and Tein conducted a study with 1362 Chinese adolescents, which revealed that suicide attempters reported experiencing negative life events more frequently than those with suicidal ideation, and non-suicidal adolescents experienced such events the least. This finding indicates a dose–response relationship between the number of negative life events and suicidal behaviors. The relationship remained significant even after accounting for the presence of internalizing/externalizing problems [41]. Another study found that individual factors, such as female gender, smoking, alcohol consumption, internalizing and externalizing problems, and hopelessness, and external factors, such as a history of suicidal behaviors among friends and acquaintances, poor family economic status, and a negative parental relationship, were all significantly associated with an increased risk of suicidal behavior [42]. It is important to note that while these factors are associated with an increased risk of suicidal behavior, they do not necessarily cause suicidal behavior, and other factors may also play a role in the development of suicidal behavior. One-third of the patients from our sample had poor socioeconomic status, and almost half of them reported negative life events, such as death of a parent or close person, divorced or separated parents, parents working abroad, school failure, conflicts with parents or peers, and family conflicts. Additionally, up to one-third of the patients had a family history of psychiatric disorders (drug or alcohol abuse, depression, or anxiety). No significant differences were reported between the studied groups (suicidal ideation, self-harm behaviors, and suicide attempts) regarding family characteristics, but that could be explained by shared clinical features, with most patients being diagnosed with depression and different comorbidities and almost half of them had a previous suicide attempt. We aimed to detect the interaction effects of environment, psychiatric diagnosis, and sex on the prediction of suicidal ideation, self-harm behaviors, and suicide attempts. However, the logistic regression analysis did not find any significant interaction effects among the predictors, indicating that the individual predictors alone were the best predictors of the clinical variables. Although our analysis did not yield any significant results, it is important to continue investigating potential interaction effects to better understand the complexity of suicidal behavior and improve prevention and intervention strategies.

This study has certain methodological limitations that need to be taken into consideration. The study design was cross-sectional, meaning that a cause-and-effect relationship between the variables under investigation could not be deducted. The size of the clinical sample was relatively small and included only emergency-admitted patients, implying a higher severity of MHPs. Other important limitation is that we did not include a control group from the general population or from the patients’ receiving services in outpatient settings or patients who were admitted without the emergency criteria or were emergency admitted but without self-harm behaviors, suicide attempts, and suicidal ideation. Additionally, the severity of depressive symptoms was not considered separately.

Suicidal behaviors and NSSIs can be influenced by cultural and contextual factors, such as social norms, stigma, and access to mental health services. Future research should consider these factors and how they interact with environmental, school, and family factors to contribute to suicidal behavior.

## 5. Conclusions

Our results suggest that there is a strong association between self-harm behaviors, suicide attempts, and externalizing pathology. Girls with depressive symptoms from our sample were more likely to have suicide attempts than boys, and girls with depressive symptoms and behavioral problems were more probable to present self-harm behaviors than boys. The sample included children and adolescents at a high risk for suicide attempts, with important MPHs, since more than half of them had comorbidities and previous suicide attempts. Therefore, it is crucial to explore the presence of concurrent internalizing and externalizing pathologies and their association with self-harm behaviors, as it could help identify those at risk for suicidal behavior. Regarding family environment, suicidal ideation and attempts and self-harm behaviors were more frequent in patients with conflictual or disorganized families, divorced parents, and family history of depression/anxiety, violence, or alcohol/drug abuse. Overall, externalizing symptoms play an important role in the development of suicidal behavior in adolescents. It is important for mental health professionals to screen for these symptoms and treat them to reduce the risk of suicide.

## Figures and Tables

**Table 1:** Sample characteristics.

	Suicide Attempt (*n* = 32)	Suicidal Ideation (*n* = 73)	Self-Harm Behaviors (*n* = 53)
Child characteristics			
Mean age (SD)	15.09 (1.86)	14.54 (2.17)	14.94 (1.79)
Gender *n* (% male)	10 (32.25)	35 (47.9)	20 (37.73)
Environment *n* (% urban)	15 (46.8)	34 (46.57)	32 (60.37)
Personal history of psychiatric disorders *n* (%)	16 (50.00)	42 (57.53)	33 (62.26)
Personal history of chronic somatic disorders *n* (%)	2 (6.25)	4 (5.47)	2 (3.77)
School performance *n* (% poor)	12 (37.5)	28 (38.35)	23 (43.39)
Negative life events *n* (%)	12 (37.5)	33 (45.20)	26 (49.05)
Psychotherapy *n* (%)	23 (74.19)	58 (79.45)	42 (79.24)
Pharmacotherapy *n* (%)	22 (70.96)	59 (80.82)	38 (71.69)
Family characteristics			
Mother’s age (SD)	42.00 (5.76)	42.02 (7.00)	42.02 (5.42)
Father’s age (SD)	45.70 (5.95)	45.88 (7.34)	45.70 (6.50)
Parents’ marital status *n* married (%)	9 (29.03)	20 (27.39)	13 (24.52)
Parents‘ employment status Employed mother *n* (%)	10 (32.25)	25 (34.24)	17 (32.07)
Employed father *n* (%)	17 (54.83)	37 (50.68)	24 (45.28)
Parents’ education *n* (%)—mother			
8 grades	11 (35.48)	25 (34.24)	16 (30.18)
High school	12 (38.70)	28 (38.35)	18 (33.96)
University	4 (12.90)	9 (12.32)	6 (11.32)
Parents’ education *n* (%)—father			
8 grades	10 (32.25)	19 (26.02)	15 (28.30)
High school	9 (29.03)	20 (27.39)	14 (26.41)
University	4 (12.90)	10 (13.69)	6 (11.32)
Socioeconomic status (SES) *n* (%)			
Low	10 (31.25)	22 (30.13)	17 (32.07)
Medium	10 (31.25)	22 (30.13)	15 (28.30)
Good and very good	11 (34.37)	26 (35.61)	18 (33.96)
Familial conflicts *n* (%)	15 (46.87)	35 (47.94)	27 (50.94)
Family history of psychiatric disorders *n* (%)	15 (46.87)	26 (35.61)	21 (39.62)

**Table 2:** Cross-tabulation of gender and environment vs. clinical variables.

		Gender			Statistics
		Boys (*n* = 45)	Girls (*n* = 50)	Total (*n* = 95)	(Boys vs. Girls/ Urban vs. Rural)
Major depressive disorder (MDD)	SUICIDE ATTEMPT	6 (13.3%)	9 (18%)	15 (15.8%)	*χ*^2^(1) = 5.00, *p* = 0.02/ *χ*^2^(1) = 0.62, *p* = 0.42
	SUICIDAL IDEATION	19 (42.2%)	19 (38%)	38 (40%)	*χ*^2^(1) = 0.74, *p* = 0.38/ *χ*^2^(1) = 1.66, *p* = 0.19
	SELF-HARM BEHAVIOR	8 (17.8%)	13 (26%)	21 (22.1%)	*χ*^2^(1) = 1.87, *p* = 0.17/ *χ*^2^(1) = 0.83, *p* = 0.36
Mixed affective disorder and conduct disorder	SUICIDE ATTEMPT	3 (6.7%)	10 (20%)	13 (13.7%)	*χ*^2^(1) = 3.32, *p* = 0.06/ *χ*^2^(1) = 0.91, *p* = 0.33
	SUICIDAL IDEATION	11 (24.4%)	14 (28%)	25 (26.3%)	*χ*^2^(1) = 0.18, *p* = 0.89/ *χ*^2^(1) = 2.40, *p* = 0.12
	SELF-HARM BEHAVIOR	8 (17.8%)	17 (34%)	25 (26.3%)	*χ*^2^(1) = 3.97, *p* = 0.04/ *χ*^2^(1) = 0.33, *p* = 0.56
Other disorders	SUICIDE ATTEMPT	1 (2.2%)	3 (6%)	4 (4.2%)	*χ*^2^(1) = 3.25, *p* = 0.07/ *χ*^2^(1) = 0.32, *p* = 0.56
	SUICIDAL IDEATION	5 (11.1%)	4 (8%)	9 (9.5%)	*χ*^2^(1) = 0.44, *p* = 0.50/ *χ*^2^(1) = 0.32, *p* = 0.56
	SELF-HARM BEHAVIOR	4 (8.9%)	3 (6%)	7 (7.4%)	*χ*^2^(1) = 0.12, *p* = 0.72/ *χ*^2^(1) = 2.23, *p* = 0.13

## Data Availability

The data presented in this study are available from the corresponding author upon requet. The data are not publicly available due to privacy and ethical considerations.
